# Implementation and outcomes of a severe acute respiratory coronavirus virus 2 (SARS-CoV-2) monoclonal antibody treatment program at an academic medical center serving a high-risk patient population

**DOI:** 10.1017/ash.2021.165

**Published:** 2021-06-24

**Authors:** Scott A. Borgetti, Alan E. Gross, Janey Kottler, Kenil Patel, Jamie Paek, Alfredo J. Mena Lora, Diane Oikle, Delisa Jeffries, Jon Radosta

**Affiliations:** 1 Department of Medicine, University of Illinois at Chicago, Chicago, Illinois; 2 Department of Pharmacy Practice, University of Illinois at Chicago College of Pharmacy, Chicago, Illinois; 3 College of Nursing, University of Illinois at Chicago, Chicago, Illinois; 4 Ambulatory Services Administration, University of Illinois at Chicago, Chicago, Illinois

*To the Editor—*Monoclonal antibodies directed against the spike protein component of severe acute respiratory coronavirus virus 2 (SARS-CoV-2) became the first treatments approved for use in outpatients when bamlanivimab received emergency use authorization (EUA) in November 2020.^1,2^ Bamlanivimab has been associated with a decrease in coronavirus disease 2019 (COVID-19)–related hospitalization in patients at high risk for disease progression.^3^ However, the logistics of identifying patients suitable for these therapies and administering them in a timely fashion is challenging.^4^ Patients must be screened using eligibility criteria outlined in the EUA, including being within 10 days of symptom onset at the time of treatment and receiving an intravenous medication in a space that is safe for patients with COVID-19.

At the University of Illinois Hospital and Health Sciences System (UI Health), a multidisciplinary team including representatives from infectious diseases, internal medicine, emergency medicine, pharmacy, ethics, and employee health quickly convened to establish a patient identification system, an outreach team, and a drug administration plan. Potential patients were identified by generating a daily electronic health record (Epic, Verona, WI) report of all positive SARS-CoV-2 tests at our institution, including UI Health employees. The report was derived from our existing SARS-CoV-2 test order form, which included key patient characteristics to screen for eligibility such as age, BMI, and date of symptom onset. Patients tested outside UI Health could be referred for treatment by their providers.

The monoclonal antibody treatment program is led by an infectious disease physician, a pharmacist, and an internist with ambulatory care delivery expertise. A small group of physicians and advanced practice providers from different specialties comprised the outreach team to call patients to offer treatment, discuss the risk and benefits, and consent. Frontline providers were made aware of the program but were not expected to arrange treatment.

To regularly offer monoclonal antibody therapy, we repurposed a gastroenterology infusion space into a SARS-CoV-2 monoclonal antibody clinic on Mondays, Wednesdays, and Fridays. Outreach team clinicians were on site to perform an initial patient assessment and to respond to adverse reactions. If patients had clinically deteriorated prior to their infusion (eg, developed hypoxemia), they were directed to the emergency department and not treated. The emergency department volunteered to serve as an infusion site for scheduled patients on the weekends in addition to offering treatment on an ad hoc basis. All eligible patients were offered treatment within 48 hours of their positive test using this model.

A database of demographics, appropriateness of use, and patient outcomes for those receiving bamlanivimab was maintained for quality assurance. A medication-use evaluation of the first 100 patients treated was conducted by reviewing these data retrospectively via REDCAP. The University of Illinois at Chicago Institutional Review Board (IRB) determined that this quality assurance evaluation was exempt from IRB approval because it was conducted as part of our routine care.

Between December 2, 2020, and March 6, 2021, 100 patients were treated with bamlanivimab at UIH. Table 1 lists patient baseline demographics and outcomes. Patients with a specific symptom-onset date documented had a median duration from symptom onset to treatment of 4 days (IQR, 4–8). Nearly half of the patients treated had 2 or more risk factors for progression to severe disease. Moreover, 5 patients required an emergency department visit within 30 days of receiving bamlanivimab, 2 of whom were hospitalized for COVID-19 progression, and no patients in the cohort were intubated or died.

**Table 1.** Baseline Characteristics and Outcomes for Patients Receiving Bamlanivimab

Characteristic	No. (N=100)
Age, median (IQR)	56 (42–64)
Sex, female	50
**Race and ethnicity**	
Black, non-Hispanic	43
White, non-Hispanic	22
Non-White, Hispanic or Latino	30
Other or unknown	5
Body mass index, median (IQR)	30.1 (26.1–37.1)
Employee of health system	10
**Location of bamlanivimab administration**	
Clinic	65
Emergency department	35
**Risk factors for progressing to severe COVID-19^a^**	
Body mass index >35	31
Chronic kidney disease	5
Diabetes	36
Immunosuppressive disease	14
Receiving immunosuppressive treatment	12
Age ≥65 y	24
**Age≥55 y with the following comorbidity**	
Cardiovascular disease	6
Hypertension	34
Chronic respiratory disease	6
Time from symptom onset to bamlanivimab administration, median d (IQR)^b^	4 (5–8)
Emergency department visit or hospitalization due to worsening symptoms of COVID-19 within 30 d after bamlanivimab infusion	5 total patients with emergency department visit or hospitalization 3 of the 5 were admitted, 2 for direct complications of COVID-19

Note. IQR, interquartile range.

a >1 risk factor was present in 48% of patients.

b 18 patients only had documentation that symptoms occurred within the prior 10 days and therefore were not included in this description.

Our institution rapidly created and implemented a monoclonal antibody treatment program using a multidisciplinary team of experts and leveraging existing clinical infrastructure. Using a small centralized team of providers to identify eligible patients and offer treatment, we reduced the need for large-scale education of frontline providers, ensured familiarity with the risks and benefits of monoclonal antibodies, and helped minimize errors during screening. The model also allowed for rapid initiation of treatment once eligible patients were identified.

Our patient population was comprised largely of racial and ethnic groups (Black and non-White Hispanic patients comprised 73% of the cohort combined) disproportionately affected by the pandemic.^5^ Because these patient populations have born a disproportionate burden of the pandemic, ensuring that they have access to these novel therapeutics is an important part of improving health equity. Our outreach team’s familiarity with SARS-CoV-2 monoclonal antibodies allowed them to answer questions and alleviate fears about a medication under EUA in real time, facilitating patient uptake. Our experience suggests that similar populations may benefit from these novel therapies if resources are made available to them.

Overall, patients treated with bamlanivimab in this real-world, racially and ethnically diverse, high-risk patient cohort represented 5 (5%) of every 100 emergency department visits or hospitalizations. The rate of emergency department visits or hospitalizations among the post-hoc, high-risk subgroup (age >65 years or BMI >35) who received placebo in the final BLAZE-1 analysis was 13.5% (7 of 52).^6^

One limitation of our program was that our quality assessment showed that 4 patients did not have documentation showing they met the complete eligibility criteria. This finding highlights the need for regular quality review to ensure optimal medication use.

The real-world effectiveness of SARS-CoV-2 monoclonal antibodies may be affected as new variants of SARS-CoV-2 become more common.^7^ In vitro studies suggest reduced neutralization of several variants of SARS-CoV-2 by available monoclonal antibodies.^8,9^ Upon publication of the final report, we quickly transitioned to using casirivimab/imdevimab based on these new data. Evaluating rapid changes in evidence, multidisciplinary collaboration, and ongoing quality assurance are essential to delivering these novel agents to the patients that are most in need.
